# The impact of MRI scanner environment on perceptual decision-making

**DOI:** 10.3758/s13428-015-0563-6

**Published:** 2015-02-21

**Authors:** Leendert van Maanen, Birte U. Forstmann, Max C. Keuken, Eric-Jan Wagenmakers, Andrew Heathcote

**Affiliations:** 1 University of Amsterdam, Amsterdam, The Netherlands; 2 Max Planck Institute for Human Cognitive and Brain Sciences, Leipzig, Germany; 3 University of Newcastle, Newcastle, Australia; 4 Department of Psychology, University of Amsterdam, Amsterdam, The Netherlands

**Keywords:** Decision-making, Response-time models, Behavioral experiments, Linear ballistic accumulator

## Abstract

Despite the widespread use of functional magnetic resonance imaging (fMRI), few studies have addressed scanner effects on performance. The studies that have examined this question show a wide variety of results. In this article we report analyses of three experiments in which participants performed a perceptual decision-making task both in a traditional setting as well as inside an MRI scanner. The results consistently show that response times increase inside the scanner. Error rates also increase, but to a lesser extent. To reveal the underlying mechanisms that drive the behavioral changes when performing a task inside the MRI scanner, the data were analyzed using the linear ballistic accumulator model of decision-making. These analyses show that, in the scanner, participants exhibit a slow down of the motor component of the response and have less attentional focus on the task. However, the balance between focus and motor slowing depends on the specific task requirements.

## Introduction

Functional magnetic resonance imaging (fMRI) is one of the most widespread methods to understand the relationship between brain and behavior. fMRI measures the brain’s local dependence on oxygenated blood, providing insights in the metabolic response to neural activity (Huettel, Song, & McCarthy, 2009). fMRI studies have increased our understanding of the brain and its relation to behavior tremendously. However, given the popularity of fMRI, it is surprising that only a few studies have examined the effects of fMRI itself on behavior (to our knowledge, the only papers that explicitly address this question are Assecondi et al., 2010; Hommel, Fischer, Colzato, van den Wildenberg, & Cellini, 2012; Koch et al., 2003; Koten, Langner, Wood, & Willmes, 2013). These studies have not led to a consensus on the effects of MRI scanner environment.

A possible reason for this lack of consensus lies in the different ways the scanner environment was simulated. Hommel et al. (2012) studied the effect of noise—as generated by echo planar imaging (EPI)—and found that performance on cognitive control tasks increased when participants were subjected to acoustic noise sequences. That is, in a series of three experiments, participants’ response times (RT) decreased on trials where more cognitive control was required relative to trials in which less cognitive control was required. Error rates displayed a similar pattern of improvement. Hommel et al. (2012) interpreted their results in terms of a stress-induced increase in attentional control (Chajut & Algom, 2003; Plessow, Fischer, Kirschbaum, & Goschke, 2011).

It is interesting to note that in the experiment by Hommel et al. (2012) the effect of acoustic noise only appeared in interaction with the cognitive control manipulation. That is, when averaged across the levels of the cognitive control manipulation, RTs as well as error rates were unaffected by hearing loud noises. This is in contrast to Koch et al. (2003), who studied the effect of a horizontal orientation of the participant in addition to noise, and found that RTs increased. In a between-subject design, participants either performed a spatial judgment task while sitting behind a standard computer screen, or while lying on a stretcher, or while inside an operating MRI scanner. Mean RTs increased from sitting upright, to lying down, to the actual scanner experience, while error rates remained unaffected. The results were interpreted in terms of response execution. Thus, post-cognitive stages of task processing were thought to be affected by the different environments, which would explain why only RTs and not error rates were affected. Similar effects were reported by Assecondi et al. (2010). In three experimental paradigms (stimulus detection, go/nogo, and simple choice), these authors found generally increased RTs inside the scanner bore (without an actual scan sequence). No error rates were reported. It should be noted that in this study several participants displayed the opposite effect: mean RTs were shorter inside the scanner. This study did not focus on the analysis of errors, and so no interpretation of these results was provided.

The apparent inconsistency of results across experimental paradigms, and even within paradigms (Assecondi et al., 2010), is increased when considering a fourth study (Koten et al. 2013). In a series of experiments including categorization, mental arithmetic, and working memory tasks, Koten et al. (2013) found that RTs were decreased when inside the scanner. Similar to Hommel et al. (2012), there was no effect on error rates. The authors interpreted their results in terms of stress-induced arousal during the scanner sessions, leading to increased performance.

### Explanations for the effect of scanner environment

The few experiments that have examined the effect of MRI scanner environment on behavior show inconsistent results, and consequently offer different explanations of the effects of scanner environment. The first explanation is in terms of motor slowing (Koch et al., 2003). Motor slowing means that participants slow down because they take more time to execute the response. This can for example occur due to unfamiliar response devices or changes in stimulus-response mapping due to orientation (Koch et al., 2003).

The second explanation is in terms of response caution. This account assumes that participants increase the control over behavior by responding more cautiously when inside the MRI scanner. Increased response caution leads to fewer errors, but at the cost of slower responses (Bogacz, Wagenmakers, Forstmann, & Nieuwenhuis, 2010; Wickelgren, 1977). The results of Koch et al. (2003) and Assecondi et al. (2010) may be consistent with this interpretation, although it predicts decreased error rates, which were not found in these studies.

The third explanation is that participants are faster inside the scanner because they are aroused by the scanner experience (Koten et al., 2013). This explanation is in some sense related to a response caution explanation, because both explanations predict improved performance of response times. However, the explanations make different predictions for speed-accuracy tradeoff behavior. The response caution explanation predicts a slow down that allows more accurate responding, whereas an arousal explanation predicts a speed-up that is possibly also accompanied by more accurate responding. The results of Hommel et al. (2012) are consistent with an arousal explanation, under the assumption that increased control particularly affects trials in which control is important, such as switch trials in a task switching paradigm (Hommel et al.’s Experiments 1 and 2) or incongruent trials in a Simon task (Hommel et al.’s Experiment 3).

The fourth explanation of the effect of scanner environment on behavior is that participants are slower in the scanner due to a decrease in attention. That is, attentional focus is diverted from the imperative stimulus, leading to slower (Assecondi et al., 2010) and potentially more erroneous responding. Table 1 summarizes the four explanations of the effect of scanner environment on behavior.

**Table 1:** Theoretical accounts of the effect of scanner environment for correct and erroneous responses, respectively

	Motor slowing	Response caution	Arousal	Attentional focus
Cognitive explanation	Motor execution time *↑*	Response caution *↑*	Attention *↑*	Focus *↓*
Response time	*↑*	*↑*	*↓*	*↑*/=
Error rate	=	*↓*	=	*↑*/=
LBA model parameter	*t*_0_ *↑*	*b* *↑*	*v*_*c*_ + *v*_*e*_ *↑*	*v*_*c*_ − *v*_*e*_ *↓*

*Arrows* indicate the change in a parameter inside an MRI scanner relative to outside. =: no change. *b*: LBA threshold parameter; *t*_0_: LBA non-decision time parameter; *v*_*c*_ and *v*_*e*_: LBA drift rate parameters

#### Response-time modeling

In order to assess and compare the adequacy of the various competing explanations for the effects of scanner environment, we analyzed three experiments with the help of response-time models. To this end, we applied response-time models to the experiments described below. Response-time models are a class of cognitive models that allow for the decomposition of RT distributions and error rates into meaningful psychological constructs (Mulder, Van Maanen, & Forstmann, 2014; Smith & Ratcliff, in press). In particular, these models hypothesize that choice behavior can best be described as a gradual accumulation of evidence for either response alternative until enough evidence has been accumulated to give a response. The response time is determined by the time required to reach this level of evidence; whether this response is correct or not also depends on this level of evidence. If the required level of evidence is low, meaning that little evidence is required to make a response, the probability of responding incorrectly increases due to noise in the evidence accumulation process; if the required level of evidence is high, the probability of a correct response increases, but more time is required for accumulation. This way, response-time models account for RT distributions and error rates in a unified way. An additional benefit of response-time models is their ability to deconstruct dependent variables in multiple latent variables. That is, multiple cognitive processes may differ across experimental conditions. Yet, their interplay may predict no effect on the level of the observed mean behavior. Response-time modeling could uncover this by taking into account the shape of RT distributions.

**Fig. 1:**
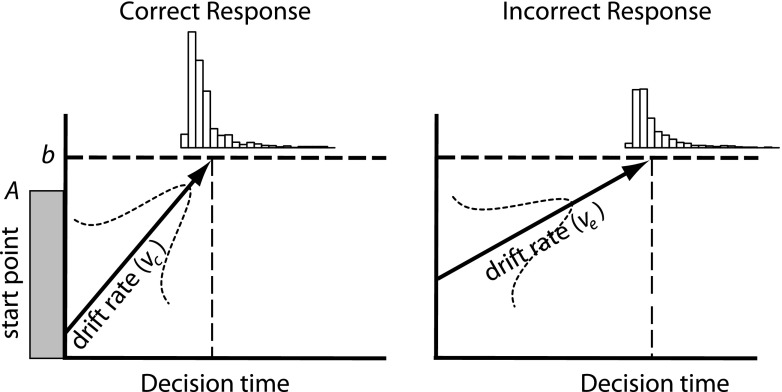
The linear ballistic accumulator model accounts for the RT distributions of correct and incorrect responses. See text for details

The linear ballistic accumulator model (LBA, Brown & Heathcote, 2008, illustrated in Fig. 1) accounts for RT distributions and error rates by estimating the following set of parameters. The average rate of evidence accumulation for a particular response alternative is represented by the *drift rate* *v*, which varies according to a Gaussian distribution from trial to trial, with a standard deviation of *sv*. Each response alternative is represented by a separate drift rate (here we use *v*_*c*_ for the correct accumulator matching the stimulus and *v*_*e*_ for the error accumulator, the accumulator mismatching the stimulus). The level of response caution is represented by the *threshold* *b*. If participants are more cautious, *b* will be higher. In some cases (e.g., Experiment 1 below), it is pertinent to assume that *b* differs between response alternatives, because participants may be more inclined to give one response over another. The level of response caution may also differ from trial to trial, which is indicated by a uniform distribution of start points of accumulation bounded by *A* and 0. Finally, processes that do no contribute to the decision are encoded in a *non-decision time* parameter *t*_0_.

The decomposition of RT and error rate in drift rates, threshold, and non-decision time provides clear predictions for the four theoretical accounts outlined above. Firstly, if non-decision times *t*_0_ are consistently higher in the MRI scanner than outside of the scanner, then this suggests that the execution of responses is hampered. This may reflect the use of different response devices inside a scanner and would be in line with a motor slowing interpretation (Koch et al., 2003, see Table 1).

Secondly, an increase in response caution would be reflected in an increased threshold parameter *b*. This parameter influences the speed–accuracy trade-off behavior of the model, decreasing error rates at the expense of RT (Bogacz et al., 2010; Wickelgren, 1977).

Thirdly, if participants are more aroused inside the scanner (Koten et al., 2013) then this is reflected in increased drift rates (Ratcliff & Van Dongen, 2011). If drift rates for all alternatives are higher, then responses will be faster, independent of which response is correct. Under this account, the LBA model predicts that the sum of drift rates *v*_*c*_ + *v*_*e*_ will increase. If the drift rate of one alternative increases more than the drift rate of the other alternative, than the arousal theory also predicts that error rates are different inside the scanner. For example, error rates will be lower if the drift rate for correct responses increases more than the drift rate for incorrect responses as compared to a behavioral experiment outside the scanner.

Fourthly, the difference between drift rates reflects the relative focus on one alternative over the other (Mulder & Van Maanen, 2013, White, Ratcliff, & Starns, 2011). If participants are better in extracting the relevant information from the stimulus, than the drift rate of the intended response is increased relative to the drift rate for the incorrect response alternative. Thus, the focus account predicts that the drift rate difference *v*_*c*_ − *v*_*e*_ is smaller inside the MRI scanner, because of a decrease in attentional focus.

### Experiments

We analyzed three experiments (two re-analyses and one new experiment) that were executed inside as well as outside the MRI scanner. All three experiments involved a random dot motion task, in which participants had to indicate the direction of the flow of motion of a cloud of pseudo-randomly moving dots (Ball & Sekuler, 1982; Palmer, Huk, & Shadlen, 2005; Forstmann et al., 2008; Van Maanen et al., 2012; Van Maanen, Grasman, Forstmann, & Wagenmakers, 2012). In the experiments the perceived motion is generated by a linear displacement of a proportion of the dots either to the left or to the right. The remaining dots move randomly. Each experiment manipulated a single property that is known to affect a specific model parameter. Experiment 1 manipulated response probability. Participants received a cue indicating the most likely direction of motion on the upcoming trial. For example the cue “L9” indicated that the motion direction would be leftward with a 90 % probability. This kind of manipulation typically biases participants to respond in the cued direction. In terms of the LBA model, this kind of behavior is best described by a decrease in the threshold parameter of the more likely alternative (Forstmann, Brown, Dutilh, Neumann, & Wagenmakers, 2010; Mulder, Wagenmakers, Ratcliff, Boekel, & Forstmann, 2012).

Experiment 2 cued participants to respond either as fast as possible, or as accurately as possible. Typically, participants are faster and more error-prone on the speed-cued trials. This trade-off between fast and inaccurate versus slower but accurate responding can be accounted for by a change in the threshold parameters of decision-making models (e.g., Bogacz et al., 2010), possibly in combination with changes in non-decision time (e.g., Rae, Heathcote, Donkin, Averell, & Brown, 2014).

Experiment 3 manipulated the difficulty of the random dot motion task by changing the proportion of coherently moving dots. That is, on the hardest trials only 5 % of the dots moved coherently to either left or right, with the remaining dots moving randomly. On the easiest trials, 80 % of the dots moved coherently. As the coherence increases and the task becomes easier, participants tend to respond faster and make fewer errors. In response-time models this is often accounted for by the drift rate parameter (e.g., Palmer et al., 2005).

The fMRI sessions of Experiment 1 and 2 have been previously published (Forstmann et al., 2010, 2008, respectively). Both experiments, however, included a behavioral session outside of the scanner, analyses of which have not been previously published. Here, we focus on the differences in behavior and model parameters between the MRI session and the behavioral session. The behavioral practice sessions were administered before the MRI session. Therefore, to exclude the possibility that any effects related to the experimental session are caused instead by session order, we conducted Experiment 3. Experiment 3 involved an “MRI” session in a mock MRI scanner and a behavioral session, administered in an order that was fully counterbalanced across participants. To study the possible effect of the timing of trials in a typical event-related fMRI experiment (inter-trial intervals are usually much longer in a scanner), Experiment 3 also featured a manipulation of inter-trial interval. Before turning to the response-time modeling we first discuss the behavioral results of all three experiments.

## Experiment 1: Choice bias

### Methods

Experiment 1 was previously published by Forstmann et al. (2010). Nineteen participants performed a random dot motion task in which each trial was preceded by a cue indicating the most likely direction of motion. The cues could either be reliable (meaning that they were valid on 90 % of the trials), moderate (valid on 70 % of the trials), or neutral (that is, no prior information is given). Because the reliable and moderate cues were valid on a subset of the trials, the experiment also includes invalid trials, on which the cues incorrectly indicated the upcoming motion direction. In total, there were, therefore, five conditions. Each participant performed a behavioral session followed by an MRI session.

The behavioral session preceded the MRI session by two days. In the behavioral session, participants completed 840 trials. Each trial started with a cue presented for 1,000 ms, followed by a cue-stimulus interval of 500 ms and the stimulus for 1000 ms. Feedback on participants’ performance was presented next for 350 ms. The MRI session consisted of 240 trials. Here, each trial started with a variable jitter of 0, 500, 1,000, or 1,500 ms, followed by the cue (4,800 ms). Next there was again a variable jitter sampled from the same set of durations, and the stimulus for 1,500 ms. Feedback was presented for 350 ms.

#### Bayesian analysis of variance

To quantify the likelihood that behavior differs across experimental manipulations, we fit an ANOVA model to mean RTs for correct responses and the error rates, and analyzed the factor of the model using a *Bayes factor* (Rouder, Morey, Speckman, & Province, 2012).^1^ The Bayes factor quantifies the odds that the observed data occurred under the null hypothesis versus an alternative hypothesis. In this case, the Bayes factor can be quantified by the weighted likelihood ratio that compares the full ANOVA model vs. a model that omits a particular factor Thus, a Bayes factor of 2 indicates that the observed data are two times as likely to be consistent with a model that included a particular factor than a model that does not include that factor.

The use of Bayesian statistical analyses permits quantification of support for the null hypothesis. Thus, if an effect is truly absent, this will be indicated a large Bayes factor (Jeffreys, 1961; Rouder et al., 2012) in favor of the null hypothesis. Conversely, if an effect is absent in the data due to low statistical power, then this will result in a Bayes factor close to 1, indicating that the data does not clearly speak in favor of the presence nor the absence of the effect.

In addition, Bayesian statistics provides a continuous measure of support of the alternative and null hypotheses allowing an interpretation that does not depend on specific cut-off criteria (general introductions to Bayesian statistical analyses are provided by Bolstad, 2007; P. Lee, 2012; M. Lee & Wagenmakers, 2013).

In Experiment 1, we fit a model with Session, Cue, and stimulus Direction as fixed factors, and Participant as a random factor. Because a leftward (rightward) cue followed by a left (right) motion is a valid cue, but a leftward (rightward) cue followed by a right (left) motion is an invalid cue, the bias effect is expected to be reflected by a Cue × Direction interaction.

The Bayes factor represents the weighted likelihood ratio that compares the full ANOVA model vs. a model that omits a particular factor (Rouder et al., 2012). Thus, if a particular experimental manipulation is not reflected by effects in the data, the marginal likelihood of the full ANOVA model will be less than the marginal likelihood of the restricted model that omits that manipulation as a factor, and the Bayes factor will be in favor of the restricted model. For brevity, we will indicate the Bayes factor of the full model against a model that omits a factor with *BF*_*factor*_. For example *BF*_*Session*_ = 10 indicates that the data are 10 times more likely under the full model (that includes Session as a factor) than under the model that omits the factor Session.

In Experiment 4, we fit a model with Session, Cue, and stimulus Direction as fixed factors, and Participant as a random factor. Because a leftward (rightward) cue followed by a left (right) motion is a valid cue, but a leftward (rightward) cue followed by a right (left) motion is an invalid cue, the bias effect is expected to be reflected by a Cue × Direction interaction.

### Results

**Fig. 2:**
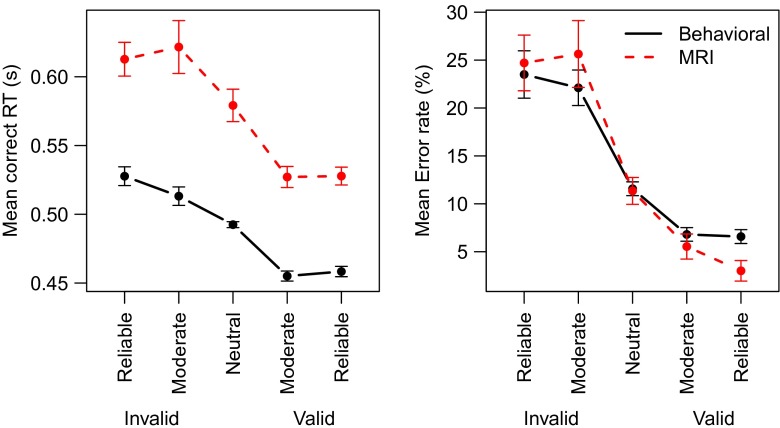
Mean behavior in Experiment 3 displays effects of cue validity and session. *Dashed red line* MRI session; *Solid black line* Behavioral session. *Error bars* represent within-subject standard errors of the mean (Loftus & Masson, 1994). In this and subsequent figures the data pertaining to the MRI sessions are indicated in *red*

The behavioral data are summarized in Fig. 2. For clarity, the figure represents the Cue × Direction interaction as valid and invalid trials, showing that correct RTs and error rates decrease as the cue becomes more reliable. This illustrates the success of the experimental manipulation. Also Fig. 2 shows that the behavioral session is on average faster than the MRI session.

These results are supported by Bayesian ANOVAs. Omitting the Session factor from the full model gives a Bayes factor of *BF*_*Session*_ = 3.9 × 10^20^ relative to the full model. This means that it is 3.9 × 10^20^ times more likely that Session had a systematic effect on the RTs than that it did not. As expected, the Cue × Direction interaction also had a strong effect, with *BF*_*Cue* × *Direction*_ = 1.2 × 10^13^. Omitting any of the other factors or interactions indicated that a reduced model was more likely than the full model, indicating that these factors did not systematically influence the RTs.

Mean error rates decreased with cue validity (*BF*_*Cue*×*Direction*_ = 1.3 × 10^28^). However, the MRI session did not seem to affect the error rate, as indicated by Bayes factors in favor of omitting the effect from the analysis (*BF*_*Session*_ = 0.08; *BF*_*Session*×*Cue*×*Direction*_ = 0.08).

### Discussion

The interaction between the direction of the cue and the stimulus direction in both RTs and error rates confirm that the experimental manipulation was successful. That is, a leftward cue followed by a leftward stimulus decreases RTs and error rates, but a leftward cue followed by a rightward stimulus increases RTs and error rates (and similar for rightward cues). This opposing mechanism explains the observed interaction between cue and stimulus direction.

Experiment 1 clearly showed slower responses for the MRI session as compared to the behavioral session, but no clear effect on the error rates. This finding seems most consistent with a motor slowing account, although both the response caution account and an attentional focus account cannot be ruled out completely. Both of these accounts are consistent with slowed down responses in the MRI session. However, a control explanation would also predict a decrease in error rates, as a consequence of exerting more control in the MRI session (Bogacz et al., 2010). In contrast, an attention account would predict an *increase* in error rate. A decrease in attentional focus would entail that the extraction of information from the stimulus is hampered, leading to more errors.

Because RTs are longer in the MRI session, a general arousal account seems unlikely. This account would predict an opposite effect for the RTs. Also, because the MRI session followed the behavioral session, an explanation of these findings in terms of practice is also ruled out. Such an interpretation would entail faster responses for the second session, which stands in contrast to the current findings.

## Experiment 2: Speed-accuracy trade-off

### Methods

Experiment 4 was previously published by Forstmann et al. (2008). Nineteen participants performed a random dot motion task in which each trial was preceded by a cue stressing either response speed or response accuracy. Specifically, on one-third of the trials participants received a cue indicating that speed was stressed, and on one-third of the trials participants received a cue indicating that accuracy was stressed. On the remaining trials the cue indicated that speed and accuracy were equally important. Participants received differential feedback depending on the condition. In the speed-stressed condition, the participants received feedback on their speed of responding; in the accuracy-stressed condition, they received feedback on whether a response was correct or incorrect; in the neutral condition, they received both types of feedback. Similar to Experiment 1 (but in contrast to Experiment 3), each participant performed a behavioral session followed by an MRI session. The timing and setup of the experiment was identical to Experiment 1.

### Results

**Fig. 3:**
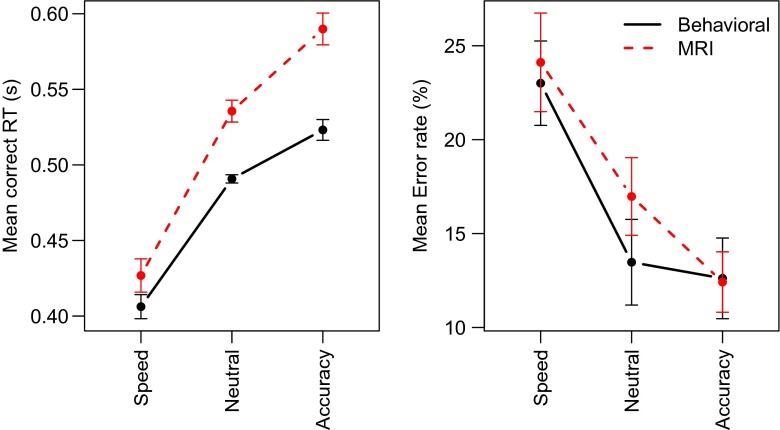
Mean behavior in Experiment 4 displays effects of cue and session. *Dashed line* MRI session; *Solid line* Behavioral session. *Error bars* represent within-subject standard errors of the mean (Loftus & Masson, 1994)

Figure 3 summarizes the results of Experiment 2. The effect of the SAT cue is evident for both correct RTs and errors, with slower responses but less errors for accuracy-stressed trials as compared to speed-stressed trials. Also, responses are slower in the MRI session than in the behavioral session.

A Bayesian ANOVA supports these observations. The ANOVA model included SAT and Session as fixed factors and Participant as a random factor. When the main effect of Session is omitted from the ANOVA model, the Bayes factor is *BF*_*Session*_ = 238. In addition, the results showed that the SAT manipulation was successful, with relatively slow responses in the Accuracy condition and relatively fast responses in the Speed condition (*BF*_*SAT*_ = 2.0 × 10^19^). There was slight evidence against an interaction effect (*BF*_*Session*×*SAT*_ = 0.38).

Similar to Experiment 1, the effect of MRI session was not clearly mirrored in the error rates, with only evidence for a successful SAT manipulation (*BF*_*SAT*_ = 6009), but moderate evidence in favor of the simpler model without the Session factor (*BF*_*Session*_ = 0.26) nor the interaction (*BF*_*Session*×*SAT*_ = 0.18).

### Discussion

Experiment 2 largely replicates the results from Experiment 1. The MRI scanner environment led to slower responses, but there is no clear analogous effect in the error rates. Again, this result mostly supports the motor slowing interpretation of the effect of MRI scanner environment advocated by Koch et al. (2003). The RT effect is also consistent with a control and attention interpretation of the data, but these accounts do make different predictions for error rates. The general arousal interpretation of the data suggested by Koten et al. (2013) does not seem to hold for this particular data set.

## Experiment 3: Task difficulty

The goal of Experiment 3 was two-fold. Firstly, we wanted to extend the results of Experiments 1 and 2 to a third experimental manipulation. Therefore, in Experiment 3 we manipulated task difficulty by adjusting the proportion of coherently moving dots. When this proportion is higher, the motion-direction is perceived more easily (e.g., Palmer et al., 2005). Secondly, we wanted to address two methodological confounds that are present in Experiments 1 and 4 because these experiments had not been designed for our particular analysis. The first confound relates to the order of the sessions. In Experiment 1 and 2 the behavioral session was intended as a training session and therefore it always preceded the MRI session. This could have led to differences between the sessions that are due to training rather than due to scanner environment. Although the data from Experiments 1 and 2 are inconsistent with a practice effect, it is nevertheless desirable to control for this confound explicitly. Therefore, in Experiment 3 we counterbalanced the sessions across participants to control order effects.

The second potential confound relates to the timing of the events within a trial. The behavioral sessions from Experiments 1 and 2 used shorter delays between cues and stimulus, and shorter inter-trial intervals (ITI) than those used in the MRI session. This was done to maximize the number of trials in the behavioral session. However, these timing differences could have led to unwanted effects on performance. To control for timing effects, in Experiment 3, we counterbalanced short and long ITIs within participants.

### Methods

The procedure of Experiment 3 was approved by the local ethics committee of the University of Amsterdam. In Experiment 3, participants had to indicate the direction of motion of a cloud of moving dots (Ball & Sekuler, 1982). The coherence of the dot cloud was determined on a trial-by-trial basis and could be either 5 %, 10 %, 20 %, 40 %, or 80 %, which were pseudo-randomly selected on a trial-by-trial basis, such that each coherence appeared equally often for each participant, session, and block. Twenty participants (15 women, mean age 22.1y) participated for course credit. Each participant performed a behavioral session and an MRI session. For the MRI session, we used a mock MRI scanner in combination with recorded EPI sequences, played at the same volume as live EPI sequences (110dB, Shellock, Ziarati, Atkinson, & Chen, 1998). The mock MRI scanner consisted of a fully functioning MRI set-up that was stripped from the magnet. Participants were asked to lie down on the scanner table that was placed inside the bore. Inside the bore, participants wore noise-canceling headphones and their head was fixated to prevent sudden head movements, identical to normal MRI protocol. Thus, the MRI scanner experience was preserved, although we did not deceive the participants into thinking the MRI scanner was real. Previous studies show that the absence of a magnetic field in the mock scanner does not have an effect on behavior (Atkinson, Renteria, Burd, Pliskin, & Thulborn, 2007; Heinrich et al. 2013, 2011), which makes the results of Experiment 3 comparable to the results of Experiments 1 and 2.

In the MRI session, the participants responded through button boxes in their left and right hand. In the behavioral session, participants used the Z and M keys on a regular keyboard. The order of the sessions was counterbalanced across participants. Each of the participants finished two blocks per session. In one block, the trials followed each other with ITIs of 500 ms. In the other block, the ITI was 4,300 ms. The order of the blocks was also counterbalanced across participants.

In each block, participants performed 200 trials, for a total of 800 trials per participant. Each trial started with a fixation cross, followed after 500 ms by the stimulus. The stimulus was presented for 1,500 ms. 500 ms after the stimulus offset feedback appeared for 200 ms, which could be either *Goed* (i.e., correct), *Fout* (incorrect), or *Geen antwoord* (no answer).

### Results

**Fig. 4:**
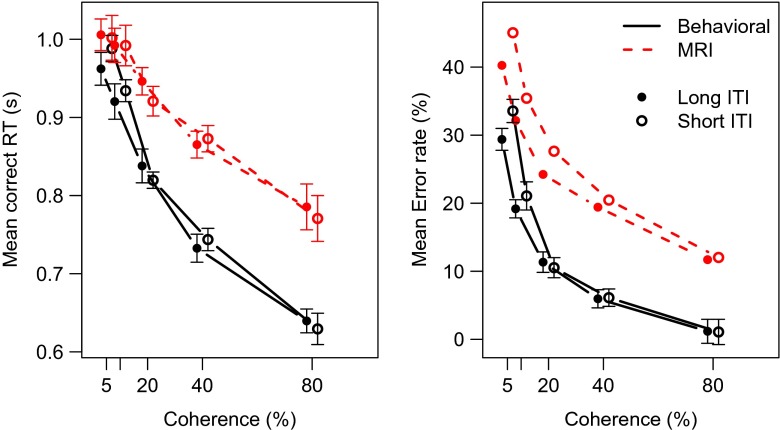
Mean behavior in Experiment 4 displays effects of stimulus coherence and session, but not of inter-trial interval. *Red lines* MRI session; *Black lines* Behavioral session. *Solid line* Long ITl; *Dashed line* short ITI. *Error bars* represent within-subject standard errors of the mean (Loftus & Masson, 1994). When not visible, the error bars fall within the area of the data point

Figure 4 summarizes the results of Experiment 3. As the coherence increases, correct RTs and error rates decrease. In addition, RTs as well as error rates are higher for the MRI session as compared to the behavioral session. The effects of ITI are not clear from Fig. 4.

The full ANOVA model that we tested included Coherence, Session, and ITI as fixed factors and Participant as a random factor. Experiment 3 replicates the increase in RT for the MRI session (*BF*_*Session*_ = 592) found in Experiments 1 and 2. Also, responses were faster for more coherent stimuli (*BF*_*Coherence*_ = 1.5 × 10^13^). However, the results show that in this data set it is not likely that the ITI contributed to the RT variance (*BF*_*ITI*_ = 0.12). Also, the Bayesian ANOVA supported a model in which all interactions were omitted (BFs < 0.14).

Contrary to Experiments 1 and 2, this experiment did show a strong effect of Session on error rate, with a Bayes factor of *BF*_*Session*_ = 4.3 × 10^18^ in favor of including the Session factor in the model. As expected, the error rates decreased with coherence, reflecting that higher coherences are easier than low coherences (*BF*_*Coherence*_ = 1.4 × 10^42^). Again, there was no effect of ITI (*BF*_*ITI*_ = 0.32), and there was no evidence for any interaction (all BFs < 0.20).

### Discussion

Experiment 3 is mostly in line with an attentional focus account of scanner environment effects. This account predicts slower responses as well as higher error rates. The response caution and motor slowing accounts are consistent with the RT data, but not with the error rate data. These accounts predict a decrease in error rate and no error effect, respectively. The arousal account is also unlikely, as that would entail faster responses and decreased error rates. One critical point is that participants were not deceived into believing that they entered a real MRI scanner. This may have affected their behavior differently than when they had entered a real MRI scanner, or when they would have been deceived. However, evidence suggest that mock MRI scanners are comparable to real scanners in terms of anxiety (McGlynn, Karg, & Lawyer, 2003; McGlynn, Smitherman, Hammel, & Lazarte, 2007), stress (Lueken, Muehlhan, Evens, Wittchen, & Kirschbaum, 2012), or noise annoyance (Pripfl, Robinson, Leodolter, Moser, & Bauer, 2006), suggesting that affects of these stressors on behavior are also comparable.

Note that in Experiments 1 and 2 the difference in ITI that was confounded with Session did not contribute to the effects observed in Experiment 3. Thus, it is unlikely that ITI is the major contribution to behavioral differences inside and outside the MRI scanner, at least for perceptual judgment tasks. This could be different for tasks in which trial dependencies are more prominent, such as the Stroop task (Juvina & Taatgen, 2009), the Simon task (Hommel, Proctor, & Vu, 2004), or related interference tasks (Botvinick, Braver, Barch, Carter, & Cohen, 2001; Gratton, Coles, & Donchin, 1992; Van Maanen & Van Rijn, 2010).

## Response-time modeling of the effect of scanner environment

All three experiments displayed slowed responses as a result of the MRI environment, but only Experiment 3 showed increased error rates. Consequently, based on mean correct RTs and error rates, the most likely candidate explanation of these effects is the attentional focus account. Because the attentional focus account assumes that the stress induced by the MRI scanner diverts attention away from the imperative stimulus, responses slow down and become more error-prone. One reason why this accuracy effect is not observed in Experiments 1 and 2 might be that another mechanism counters the increase in erroneous responses. That is, if participants are less focused, they might respond to the potential behavioral deterioration with an increase in response caution. In terms of the LBA model, this means an increase in the threshold parameter.

This hypothesis is scrutinized using formal LBA modeling of the RT distributions for both correct and error responses and error rates. First, we present our general approach for obtaining the best set of model parameters (in terms of different way of equating parameters across conditions). Next, we present the results of this analysis and what that means for the parameter estimates.

### Methods

For each experiment, we defined the *top model* as the model in which all parameters were allowed to vary across sessions (Behavioral and MRI; some exceptions are discussed below). In addition, the top models only allowed those parameters to vary across other experimental conditions for which there is consensus in the literature. For example, to account for directional cueing in Experiment 1 we allowed the threshold parameter in LBA to vary between response alternatives (cf. Forstmann et al., 2010. This parameter allows for a difference in the amount of evidence that is required for a response between the choice options, similar to the difference in prior probability that is induced in Experiment 1.

Using the top model, we generated a model hierarchy of simpler models (Heathcote & Love, 2012). That is, we generated all possible models that contained fewer free parameters by fixing a parameter across conditions. Thus, the simplest models that were fit estimate the same parameters of each type for all conditions. The best fitting parameter values of the simplest models were used as initial guesses for more complex models. This way, we reduce the impact of local minima in the parameter space. The models were fit using maximum likelihood (Donkin, Brown, & Heathcote, 2011).

The best model across participants was determined using AIC (Akaike, 1974). AIC is a measure that balances the fit of a model with the number of free parameters. The number of free parameters is a proxy for model complexity, as models with more parameters are inherently more flexible. Because of this, in a model hierarchy more complex models necessarily fit better (e.g., Collyer, 1985; Myung & Pitt, 1997). Thus, AIC allows for inferences on which parameter should be kept free across experimental conditions and which parameters should not. To determine the best model, we summed the AIC score of individual participants, and computed the AIC weights of each model (Wagenmakers & Farrell, 2004). This reflects the probability that a particular model is the best model of the set of fitted models (given the data).

In addition to AIC, we also computed Bayesian Information Criterions (BIC; Schwarz, 1978). BIC is typically more conservative than AIC in allowing model complexity. This was also observed in our model comparisons; nevertheless, the results of the BIC analyses generally agreed with the AIC analyses. For this reason, we focus our analysis on the AIC based results, and will only briefly mention any deviations from these according to BIC.

We performed Bayesian analysis of variance—similar to the analyses on the behavioral data—on the parameters of the top model. This analysis allows us to infer whether a parameter was free because of differences due to the experimental manipulations, or whether individual differences between participants forced AIC model selection to allow a free parameter, even though the variance in the estimates is not systematic with respect to conditions. The parameter estimates used in this analysis were a weighted average of estimates from all of the different model parameterizations we fit obtained using model averaging (Hoeting, Madigan, Raftery, & Volinsky, 1999; Raftery, 1995). We could have first selected one model as the *true* model for all participants, based on a criterion like having the smallest total AIC and analyzed the parameter estimates from only that model. However, this fails to take account of all of the available information (e.g., the total AIC might not be much larger for some other models, making selection of just one model questionable) and individual differences (e.g., different models maybe better for different individuals). Instead, we used the AIC values for each model to weight (Wagenmakers & Farrell, 2004) the contribution of their parameters to the overall estimates used in the Bayesian analysis of variance. The resulting estimates were fairly close to those of the model with the best overall AIC but better take into account uncertainty in the model selection process.

#### Experiment 1: Choice bias

The top LBA model allowed all five model parameters to vary across sessions. Thus, the drift rate *v*, non-decision time (*t*_0_) and *b* − *A* as well as the variance parameters *A* and *sv* were free to vary across sessions. We estimated the difference between the upper bound of the start point distribution and the threshold (*b* − *A*) to ensure that the threshold *b* could not be below the start point. Because LBA estimates separate drift rates for correct and incorrect response accumulators, *v* and drift rate variance *sv* were also allowed to vary with the type of response (that is, correct vs. incorrect). In addition, we allowed *b* − *A* to vary per accumulator as a function of the cue type (left vs. right), in line with previous models estimating bias effects (Forstmann et al., 2010).

#### Experiment 2: Speed-accuracy trade-off

As for Experiment 4, the top LBA model allowed all model parameters to vary across sessions. Also similarly, separate drift rate parameters (both mean drift rate *v* and the standard deviation of drift rate *sv*) were estimated for correct and incorrect responses. In addition, the threshold, drift rates, and non-decision time were all free to vary with the speed-accuracy manipulation. Many studies show that speed-accuracy trade-off behavior can be obtained through different threshold values (e.g., Boehm, Van Maanen, Forstmann, & Van Rijn, 2014; Forstmann et al., 2008; Mulder et al., 2010; Van Maanen et al., 2011; Winkel et al., 2012). However, some studies also show different parameter estimates for non-decision times (Heitz & Schall, 2012; Mulder et al., 2013) or drift rates (Dambacher & Hübner, 2014; Heitz & Schall, 2012; Ho et al., 2012; Mulder et al., 2010; Rae et al., 2014).

#### Experiment 3: Task difficulty

Again, all model parameters were free to vary across sessions. Because there was no credible effect of ITI (see Results), the top LBA model for Experiment 3 did not include the ITI as a factor on all parameters. Instead, we allowed only the threshold parameter to vary with the ITI. Based on the literature (Churchland et al., 2011; Donkin, Averell, Brown, & Heathcote, 2009; Ho, Brown, & Serences, 2009; Mulder et al., 2013; Palmer et al., 2005), we allowed drift rate (mean and standard deviation) to vary with coherence.

### Results

#### Experiment 1: Choice bias

**Fig. 5:**
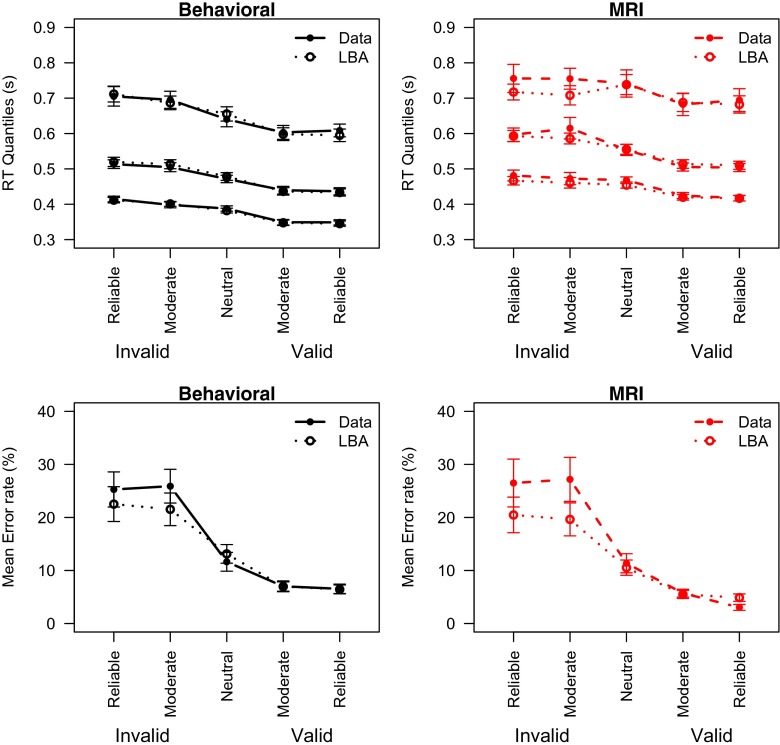
The LBA model fits the data of Experiment 3 well. *Upper panels* .1, .5, .9 RT quantiles for correct responses; *Lower panels* Error rates. The data as well as the model predictions are averaged across participants

AIC model selection preferred the following model with an AIC weight of *w*_*AIC*_ = 1.0: Threshold was allowed to vary with the cue, drift rate was allowed to vary with session and response type (correct/incorrect), and non-decision time was allowed to vary with session only. Thus, these free parameters contributed to the explanation of the data, according to AIC.^2^ Fig. 5 shows that this model indeed captures both accuracy as well as the RT distribution. Allowing a parameter to be free across conditions does not necessary entail that it will also systematically differ across participants. To test this, we submitted each parameter to a Bayesian ANOVA. The factors that were included in the ANOVA model depended on the conditions for which a free parameter value was estimated, but in all cases included the sessions. Here, we focus the analysis on the likelihood that a parameter differed per Session. In particular, we focus our analysis on the threshold parameter *b*, the non-decision time parameter *t*_0_, and the sum and difference of the drift rates for correct and incorrect responses, *v*_*c*_ + *v*_*e*_ and *v*_*c*_ − *v*_*e*_, respectively.

**Fig. 6:**
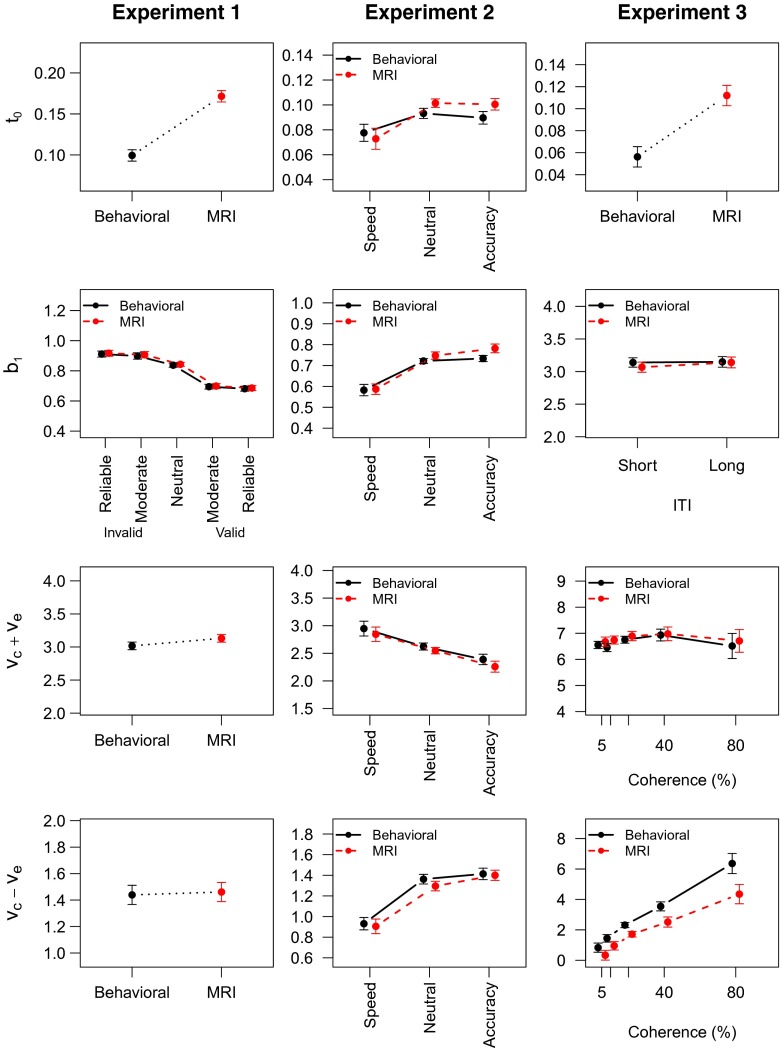
LBA model parameters for the best models for Experiments 1–3. *Left column* Experiment 1; *Middle column* Experiment 2; *Right column* Experiment 3. *Top row* non-decision time *t*_0_, representing delayed response execution; *second row* threshold of the correct accumulator *b*_*1*_, representing response caution; *Third row* sum of drift rates *v*_*c*_ + *v*_*e*_, representing overall arousal; *Bottom row* drift rate difference *v*_*c*_ − *v*_*e*_, representing attentional focus. *Error bars* are within-subject standard errors of the mean

These four parameters represent the four theoretical accounts that we focus on. Fig. 6 (Left column) shows the mean parameter estimates across participants representing the four theoretical accounts. Table 2 shows Bayes factors for omitting the crucial Session factor from an ANOVA model. For Experiment 1, there is evidence that the non-decision time parameter *t*_0_ varied with Session, as the Bayes factor for including it in the ANOVA model is quite large. For the remaining parameters *b*, *v*_*c*_ + *v*_*e*_ and *v*_*c*_ − *v*_*e*_ the evidence for including the Session factor is inconclusive.^3^ The threshold parameter did show evidence in favor of including the cue factor in the ANOVA model (*BF*=1.3×10^11^), consistent with previous studies that induced a bias in responding (Forstmann et al., 2010; Mulder et al., 2012). Also, the start point variability parameter (*A*) showed evidence in favor of a cue-related effect (*BF*=681), indicating that variability due to the distance towards the threshold was smallest for neutral cues, and larger for both valid and invalid cues.

**Table 2:** Bayes factors in favor of an LBA parameter effect in Experiment 1–3

	*t*_0_	*b*	*v*_*c*_ + *v*_*e*_	*v*_*c*_ − *v*_*e*_
Experiment 1: Bias	**381**	–	0.69	0.25
Experiment 2: SAT	0.29	1.06	0.56	0.21
Experiment 3: Difficulty	**6.2**	0.26	0.22	**75**

Bold face indicate strong evidence in favor of including the Session factor in the ANOVA model

Based on the results of Experiment 1, the most likely explanation is that participants increase the amount of motor control they exert on responding while inside the MRI scanner. This is reflected by an increased non-decision time parameter, resulting in slower yet equally accurate responses.

#### Experiment 2: Speed-accuracy trade-off

**Fig. 7:**
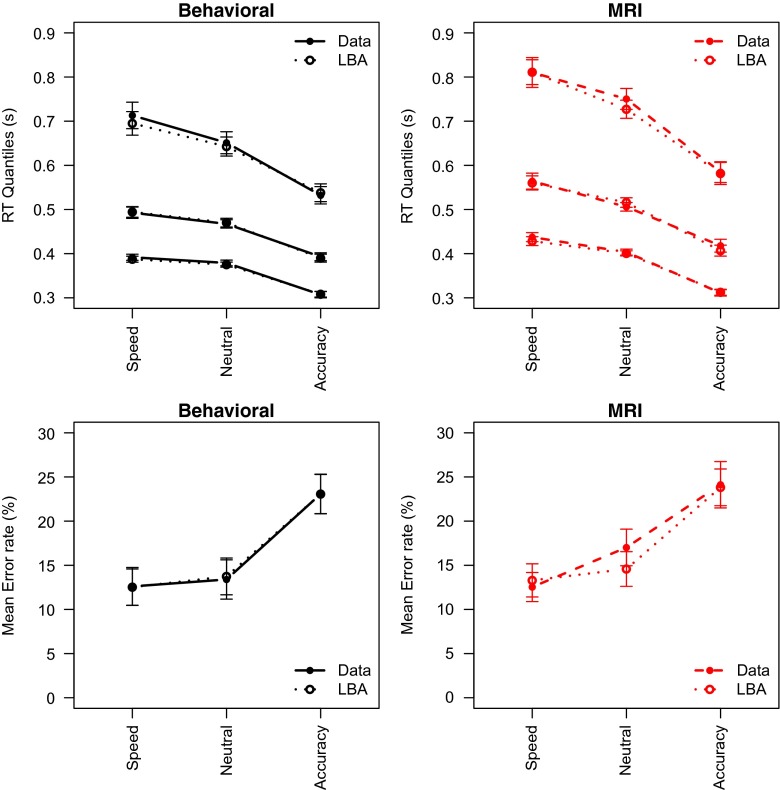
The LBA model fits the data of Experiment 2 well. *Upper panels* .1, .5, .9 RT quantiles for correct responses; *Lower panels* Error rates. The data as well as the model predictions are averaged across participants

AIC model selection on the LBA model hierarchy showed that, in order to best balance model complexity and fit across the whole set of participants, the threshold and non-decision time should be allowed to differ for the two sessions as well as the variability parameters *A* and *sv* (AIC weight of this model *w*_*AIC*_ = 1.0). Also, the threshold and drift rate parameters should be allowed to vary as a function of the SAT manipulation, and drift rate (mean and standard deviation) should differ for correct and incorrect response accumulators. This model provided a good fit to the data (Fig. 7).^4^ Fig. 6 (middle column) shows the mean parameter estimates across participants, and Table 2 again shows Bayes factors for omitting the Session factor from an ANOVA model. Omitting the Session factor from the ANOVA model yielded no conclusive evidence for any of the theoretical accounts.

The SAT manipulation did influence the estimate of the threshold (*BF*_*SAT*_ = 6.3 × 10^21^, but again there was no interaction (all BFs < 0.20). This finding is in line with previous work that reports that SAT involves adjustments in response caution (e.g., Boehm et al., 2014; Forstmann et al., 2008; Mulder et al., 2010; Van Maanen et al., 2011; Winkel et al., 2012). However, in addition to a change in threshold, there were also effects of SAT on the drift rates. In particular, the drift rate difference, which according to AIC could only be affected by the SAT manipulation, was indeed higher on accuracy trials relative to speed trials (*BF*_*SAT*_ = 1.2×10^19^). This finding is in agreement with recent literature reporting similar results (Dambacher & Hübner, 2014; Heitz & Schall, 2012; Ho et al., 2012; Mulder et al., 2010; Rae et al., 2014). Finally, there is also evidence for including separate non-decision time parameters for the three SAT conditions (*BF*_*SAT*_ = 40). Previous studies also report that non-decision time parameters may differ between speed and accuracy instructions (Heitz & Schall, 2012; Mulder et al., 2013).

#### Experiment 3: Task difficulty

**Fig. 8:**
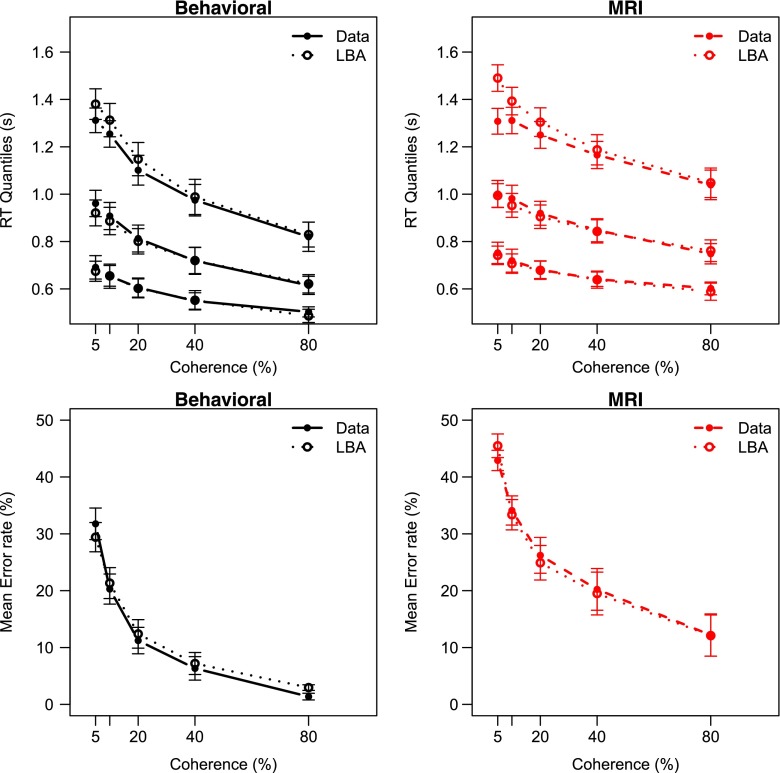
The LBA model fits the data of Experiment 4 well. *Upper panels* .1, .5, .9 RT quantiles for correct responses; *Lower panels* Error rates. The data as well as the model predictions are averaged across participants

The LBA model that was preferred by AIC was a model in which threshold varied with ITI and session, mean drift rate varied with session and coherence, and non-decision time varied with session only (*w*_*AIC*_ = 1.0). As is common for LBA models, mean drift rate as well as the standard deviation varied depending on whether a response was correct or not.^5^ The fit of this model is generally quite good (Fig. 8). The mean parameter estimates are presented in the right column of Fig. 6.

According to a Bayesian ANOVA, the non-decision parameter was higher in the MRI session than in the behavioral session (BFs = 6.2, see also Table 2). The threshold parameter did not differ between the sessions, the ITIs, nor the interaction (BFs < 0.32, see also Table 2). Neither did the sum of drift rates (all BFs < 0.22). The drift rate difference however increased with both the coherence as well as the Session (*BF*_*Coherence*_ = 7.3 × 10^22^; *BF*_*Session*_ = 75). There was no evidence in favor of an interaction between these effects (*BF*_*Coherence*×*Session*_ = 0.21).

The decrease in drift rate difference that we found is consistent with an attentional focus explanation of the effects of scanner environment. That is, if attentional focus is less, then the extraction of information from the stimulus is negatively affected, which is reflected in decreased drift rates.

The increase in drift rate difference with coherence is consistent with the literature (Churchland et al., 2011; Donkin et al., 2009; Ho et al., 2009; Mulder et al., 2013; Palmer et al., 2005). That is, as the motion direction becomes more recognizable, the extraction of information from the stimulus in favor of the correct alternative increases. Consequently, the difference in drift rates between the correct and the incorrect alternative increases as well.

In agreement with the RT and accuracy analyses, the model parameters did not differ between the first and the second session, neither in isolation (all BFs < 0.75) nor in interaction with the session type (all BFs < 1.14). Thus, the conclusion that there is no appreciable practice effect from the first to the second session seems justified. This result adds justification to the interpretation of Experiment 1 and 2, since it is likely that there too is no large effect of practice across session orders.

## General discussion

In this paper we report behavioral analyses and response-time modeling of three experiments, focused on the differences between sessions inside and outside the MRI scanner. The behavioral analyses show that participants respond slower during scanner sessions (Experiments 1–3), sometimes in relation to more errors (Experiment 3). Based on the response-time modeling of the data of all three experiments, it seems that behavioral differences between sessions inside and outside of the MRI scanner are driven by slower motor execution times (Experiments 1 and 3), sometimes in combination with a decrease in attentional focus inside the MRI scanner (Experiment 3).

Although the LBA modeling of Experiment 2 was not conclusive, this combination of mechanisms is also consistent with the behavioral pattern observed in Experiment 2. Experiment 2 displays RT slowing and no effect on error rate inside the MRI scanner. The LBA model explains this data by a combination of small effects, none of which is strong enough by itself to have strong evidence. In particular, the parameter estimates for non-decision time are slightly higher for the MRI session (Fig. 6).

It should be noted that although an explanation of these effects in terms of motor slowing is plausible and consistent with previous findings (Koch et al., 2003), there are other possible interpretations as well. Firstly, increases in non-decision time estimates are theoretically linked to time that is not spent on the decision process. Although in the current study we advocate a post-decision increase in RT—non-decision time is larger due to motor response slowing—effects on RT could also be due to increases in pre-decisional stage durations. For example, a increase in the time required for perceptual encoding of the stimulus (without a change in the probability of success of that process) would also yield a larger non-decision time. Although typically included in the non-decision time component in theoretical studies, pre-decision stages are almost never considered when fitting accumulator models to data (but see Van Maanen, Van Rijn, & Taatgen, 2012, for an exception). More research is needed to distinguish pre- from post-decision stages in the analysis of response times.

Secondly, the results may be partly attributed to a difference in lag from the actual motor response to the logging of that response by the experiment computer. That is, the use of different hardware between sessions might partly be responsible for a difference in the logged RT, which would show as a different in the non-decision time parameter. We deem this explanation unlikely however. The typical lag of a PC keyboard (USB or PS/2) is ∼20 ms (Plant & Turner, 2009). In order to explain the effect sizes on *t*_0_ that are observed in Experiment 1 the lag of a response box used in the MRI scanner would have to be in excess of ∼70 ms. Given the sub-millisecond precision of optical timing devices used in MRI setups, this is extremely unlikely, even if the differences in physical cable length is considered.

It is perhaps surprising that there are such marked differences between the experiments, but it aligns well with the general picture of the plethora of effects reported in the literature. In this case, given the overall similar nature of the three experiments we analyzed, it seems likely that multiple mechanisms are at play in each experiment. However, the relative contribution of each to the observed behavior may differ between experiments. That is, in some experimental paradigms the effect of decreasing focus might outweigh the effect of increased motor control. This seems to hold in particular for Experiment 3. Here, a decrease in attentional focus has a differential effect on each condition, with a larger impact on the difficult conditions (with lower coherence) than on the easy conditions (high coherence). In Experiments 1 and 2, the random-dot kinematograms are not so difficult that a potential decrease in focus leads to strong effects on error rates, resulting in much smaller effects on *v*_*c*_ − *v*_*e*_.

The extent to which our results generalize to other imaging modalities or experimental paradigms is not clear. Firstly, the motor slowing that was the most prevalent effect of the MRI scanner environment was not of the same magnitude across experiments. It is therefore plausible that this estimate is also not stable across different experimental paradigms. Secondly, since we did not isolate the contributing factors that make up the MRI scanner environment, it is unclear how behavior would differ from behavioral sessions in for example a magneto-encephalogram or a combined electro-encephalogram fMRI experiment. However, following Koch et al., (2003), it seems that motor slowing is related to body posture in relation to the response device. Therefore, any neuro-imaging study that affects these factors should take tour findings into account.

The results of our study are important for the field of model-based neuroscience (Mulder et al., 2014). From the response-time modeling, it is clear that model parameters are affected by the scanner environment. However, it is also clear that there are no interaction effect between Session and the experimental manipulations in any of the model parameters. Thus, even though the scanner environment affects behavior, it does so equally for all conditions in the three experiments we analyzed. This is reassuring as it shows that behavioral findings and MRI findings are comparable.

Nevertheless, it would be unadvisable to determine model parameters based on behavioral sessions alone. Rather, we suggest to model both the behavioral session and the MRI session simultaneously. This allows for greater power in determining the model parameters because of more observations, and also allows inspection of possible scanner effects.

In conclusion, we note that the effects of a scanner environment on behavior are not yet fully understood. A good approach to unraveling these is model-based analyses that uncover the underlying mechanisms of behavior. Here, we have shown that multiple mechanisms interact to cope with a different experimental environment. Future research should disentangle the sources of the experimental environment that affect these mechanisms, such as stress, noise, spatial orientation, or response mode.

## Footnotes

1 We used the BayesFactor package in R (Morey & Rouder, 2012).

2 The BIC result was similar, except that the best model according to BIC constrained the mean drift rate to only vary with response type.

3 In fact, the variability of *b* across sessions was so small that a Bayes factor could not be computed using the BayesFactors package.

4 As for Experiment 1, BIC is in agreement with AIC with respect to the Session factor, although the additional constraints are that the threshold parameters *b* and *A* should not vary with SAT, yet the non-decision time should.

5 BIC was again a bit stricter, with no effect of Session on *b*, *A*, nor *v*, but an effect of session on *sv*.
